# *Siraitia grosvenorii* Extract Protects Lipopolysaccharide-Induced Intestinal Inflammation in Mice via Promoting M2 Macrophage Polarization

**DOI:** 10.3390/ph17081023

**Published:** 2024-08-04

**Authors:** Huining Wu, Mengru Guo, Linlu Zhao, Jin Zhang, Jieyi He, Anning Xu, Zhichao Yu, Xingbin Ma, Yanhong Yong, Youquan Li, Xianghong Ju, Xiaoxi Liu

**Affiliations:** 1 Department of Veterinary Medicine, College of Coastal Agricultural Sciences, Guangdong Ocean University, Zhanjiang 524091, China; wuhuining1014@163.com (H.W.); g15638731180@163.com (M.G.); zhaolinlu@stu.gdou.edu.cn (L.Z.); zhangjin30@stu.gdou.edu.cn (J.Z.); hejieyi@stu.gdou.edu.cn (J.H.); yujingmary@163.com (Z.Y.); mxb1984612@gdou.edu.cn (X.M.); yongyanhong-007@163.com (Y.Y.); youquan-li@163.com (Y.L.); juxh@gdou.edu.cn (X.J.); 2 School of Health Science and Engineering, University of Shanghai for Science and Technology, Shanghai 200093, China; 2135070709@st.usst.edu.cn

**Keywords:** SGE, intestinal inflammation, M1/M2 polarization, RAW264.7, mice

## Abstract

*Siraitia grosvenorii* has anti-inflammatory, antioxidant, and immune-regulating effects, while macrophages play an important role in reducing inflammation. However, it is still unclear whether *Siraitia grosvenorii* extract (SGE) is effective in reducing inflammation by regulating macrophages. This study investigated the regulatory effect of SGE on macrophage polarization in a lipopolysaccharide (LPS)-induced intestinal inflammation model after establishing the model in vitro and in vivo. The results from the in vivo model showed that, compared with the LPS group, SGE significantly improved ileal morphology, restored the ileal mucosal barrier, and reduced intestinal and systemic inflammation by increasing CD206 and reducing iNOS proteins. In the in vitro model, compared with the LPS group, SGE significantly reduced the expression of iNOS protein and cytokines (TNF-α, IL-1β, and IFN-γ) while significantly increasing the protein expression of CD206 in RAW264.7 cells. In conclusion, SGE can alleviate intestinal inflammation, protect the mucus barrier, and block the systemic immunosuppressive response by increasing M2 macrophages.

## 1. Introduction

Lipopolysaccharide (LPS), a pathogenic component of the outer membrane of Gram-negative bacteria, stimulates innate immunity and causes intestinal injury and excessive inflammatory responses [1]. It induces acute inflammatory reactions in many infectious diseases, including enteritis and respiratory infections [2]. Macrophages are specialized cells in the immune system that play an important role in regulating the body’s inflammatory response [3]. The polarization state of macrophages affects the inflammatory cascade response [4]. The two most typical macrophage polarization phenotypes are the pro-inflammatory (M1) macrophage phenotype and the proliferative (M2) macrophage phenotype [5]. An imbalance in M1/M2 macrophage polarization worsens inflammation [1]. Therefore, regulating the M1/M2 polarization balance of macrophages is an effective strategy for treating intestinal inflammatory diseases.

*Siraitia grosvenorii*, a plant in the cucurbitaceae family, contains triterpene saponins, xanthones, and polysaccharides [6]. It is widely used as a non-nutritional sweetener and flavor enhancer in China, the United States, and Japan [7]. *Siraitia grosvenorii* extract (SGE), a non-toxic substance, is known for its anti-inflammatory, antioxidant, antidiabetic, and immune-regulating properties [8,9,10]. SGE is known to reduce atopic dermatitis by regulating immune dysfunction and airway inflammation in mice [11]. However, it is unclear whether SGE is effective in reducing intestinal inflammation in mice and the underlying mechanism. Therefore, this paper aims to explore the protective effect and mechanism of SGE on LPS-induced intestinal inflammation.

## 2. Results

### 2.1. Chemical Component Analysis of SGE

**Figure 1:**
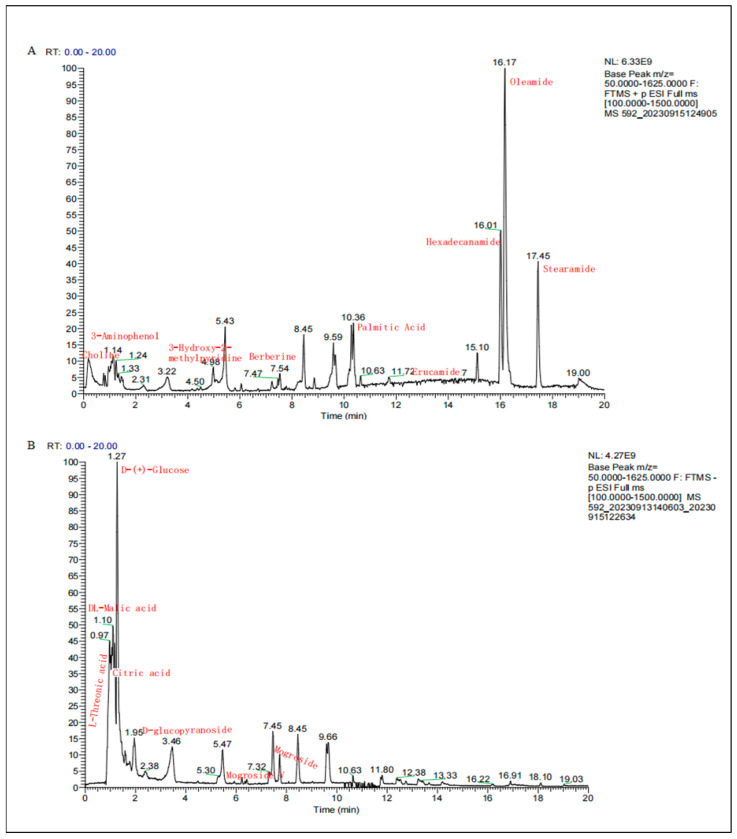
Total ion chromatogram of LC-MS analysis of SGE in positive ion mode (**A**) and negative ion mode (**B**). Note: A: Oleic acid amide, retention time 16.17 min; hexadecarbonamide, retention time 16.01 min; stearamide, retention time 17.45 min; N-methyl-anthranilic acid, retention time 5.1 min; erucic acid amide, retention time 13.41 min; palmitic acid, retention time 10.3 min; 3-aminophenol, retention time 1.14 min; choline, retention time 0.99 min; fenugreek hydrochloride, retention time 1.03; 3-hydroxy-2-methylpyridine, retention time 4.98. B: Malic acid, retention time 1.07 min; citric acid, retention time 1.16 min; glucose, retention time 1.27 min; L-threonic acid, retention time 0.88 min; mogroside, retention time 6.27–8.2 min.

The chemical constituents of SGE were identified and analyzed using LC-MS/MS to obtain information on molecular weight, relative amount, and relative concentration. In Figure 1, the retention time of different chemicals in the chromatogram represents the relative amount, and the height of the peak reflects the relative concentration of bioactive chemicals. A total of 88 chemical components were identified in SGE (Supplementary Table), including fatty acids, organic acids, sugars, and triterpenoid glycosides. The major chemical components in SGE and their relevant information are shown in Table 1. In Figure 1A, 35 compounds, mainly fatty acids, were isolated in the positive ion mode, with oleic acid amide, cetyl amide, stearic acid amide, erucic acid amide, and palmitic acid ranking in the top 5 (Table 1). As shown in Figure 1B, 53 compounds were identified in the negative ion mode, with the main ingredients being triterpene glycosides (mogroside, relative content 1.27%), sugars (glucose), and organic acids (malic acid and citric acid).

### 2.2. Effect of SGE on Blood Indices of Mice

**Figure 2:**
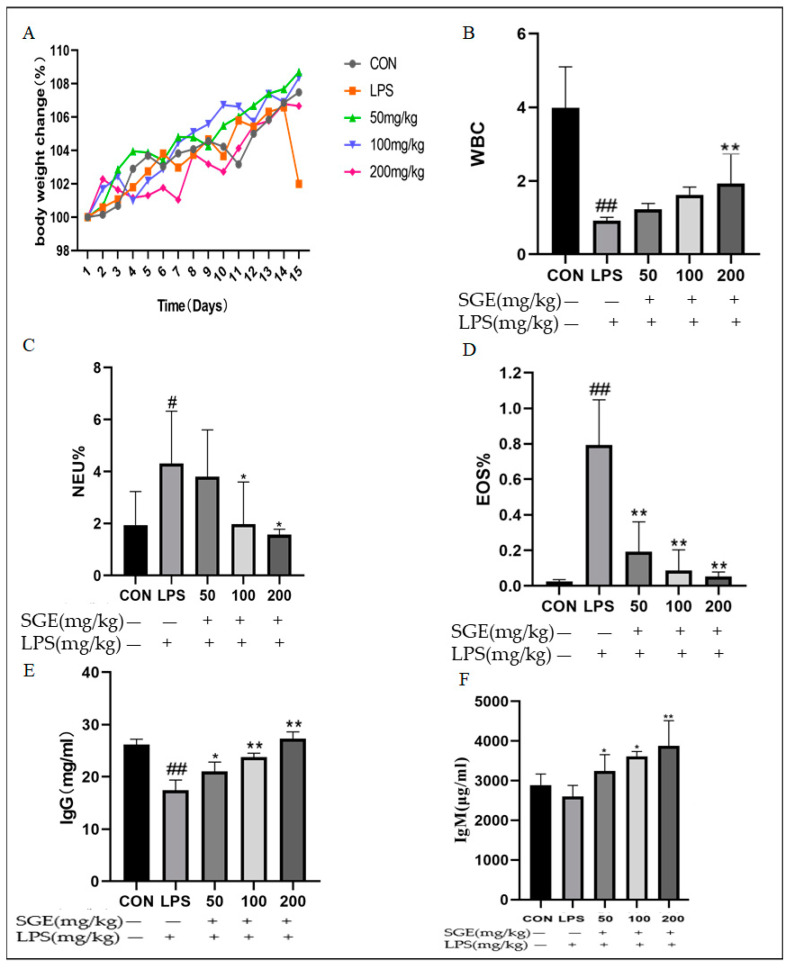
Effect of SGE on LPS-induced inflammation in mice. Changes in weight (**A**), white blood cell count (**B**), neutrophil percentage (**C**), eosinophil percentage (**D**), serum IgG levels (**E**), and IgM levels (**F**) were measured using an ELISA (*n* = 6). The results showed that compared with the CON group, # *p* < 0.05, ## *p* < 0.01. Compared with the LPS group, * *p* < 0.05, ** *p* < 0.01.

As shown in Figure 2, LPS significantly reduced the weight of the mice (Figure 2A) and the white blood cell count (*p* < 0.01, Figure 2B), while it significantly increased the percentage of neutrophils (*p* < 0.05, Figure 2C) and eosinophils (*p* < 0.01, Figure 2D). An ELISA detected changes in immunoglobulins (Ig) in mouse blood; LPS significantly reduced IgG (*p* < 0.05, Figure 2E) and IgM (Figure 2F). Compared with the LPS group, SGE (50–100 mg/Kg) increased the body weights of the mice, significantly increased the number of white blood cells (*p* < 0.01, Figure 2B), significantly reduced the percentage of neutrophils (*p* < 0.05, Figure 2C) and eosinophils (*p* < 0.01, Figure 2D) in peripheral blood, and significantly increased the content of IgG and IgM (*p* < 0.05, Figure 2E,F). The results showed that SGE improved the immune imbalance in mice with LPS-induced enteritis.

### 2.3. Effects of SGE on Ileum Histopathology in Mice with LPS-Induced Enteritis

**Figure 3:**
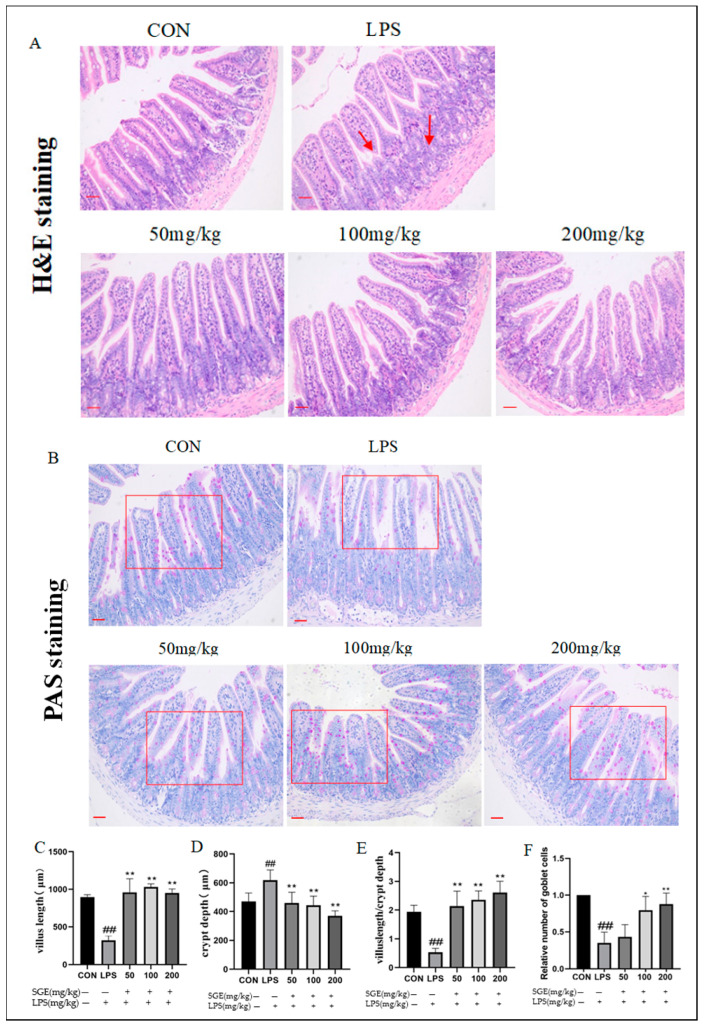
Pathological damage of ileal tissue in each group. H&E (**A**) and PAS staining (**B**); images obtained at 200×. The length of villi (**C**); the depth of crypts (**D**); the villus length/crypt depth ratio (**E**); and the relative number of goblet cells (**F**) in the mouse ileum. The scale bar is 100 μM (*n* = 6). The results showed that compared with the CON group, ## *p* < 0.01. Compared with the LPS group, * *p* < 0.05, ** *p* < 0.01. Arrows indicate villus atrophy and increased crypt depth. The box represents the area for goblet cell counting.

Compared with the control group, the LPS group had damaged ileal tissue, with more atrophy and rupture of villi (Figure 3A), sparser distribution of goblet cells (Figure 3B), and a significant decrease in the relative number of cells (*p* < 0.01, Figure 3F). The length of intestinal villi in the LPS group significantly decreased (*p* < 0.01, Figure 3C), and crypt depth significantly increased (*p* < 0.01, Figure 3D). The ratio between the length of intestinal villi and crypt depth significantly decreased (*p* < 0.01, Figure 3E). After the oral administration of SGE, the ratio between the length of intestinal villi and crypt depth significantly improved (*p* < 0.01, Figure 3E), and the number of goblet cells increased (*p* < 0.05, Figure 3F). Therefore, SGE treatment restored LPS-induced ileal tissue injury.

### 2.4. Protective Effect of SGE on Ileal Mucus Barrier of Mice

**Figure 4:**
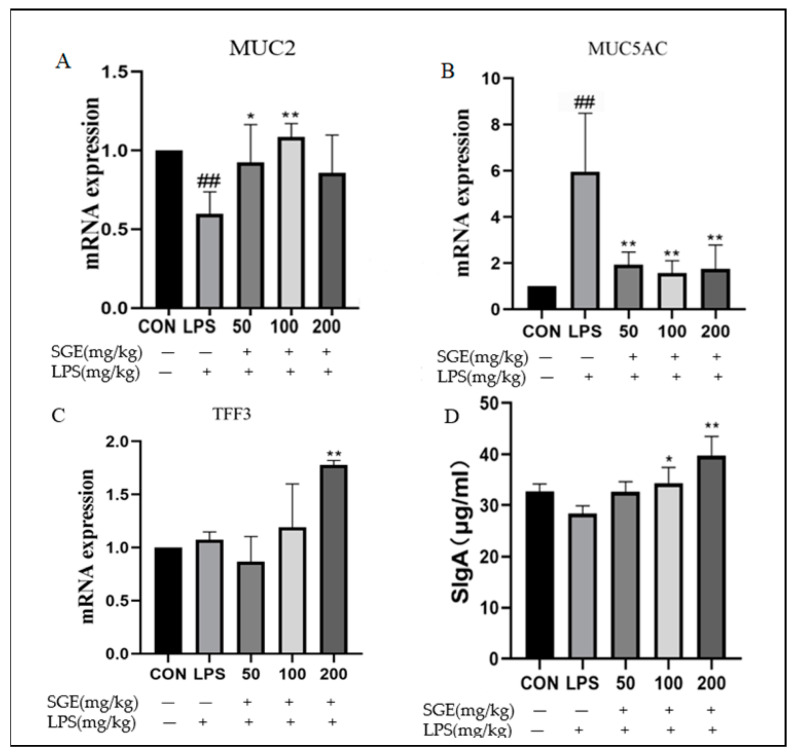
SGE alleviated LPS-induced injury to the mucus barrier. Real-time fluorescence quantitative PCR was used to detect the mRNA expression of mucin 2 (**A**), mucin 5AC (**B**), and trefoil factor 3 (**C**) in ileal tissue. ELISA was employed to detect the content of sIgA (**D**). *n* = 6. Compared with the CON group, ## *p* < 0.01. Compared with the LPS group, * *p* < 0.05, ** *p* < 0.01.

The mucus layer, including the MUC2 and MUC5AC proteins, can protect the intestinal tract from damage. MUC2 mucin forms the backbone of intestinal mucus, covering and protecting the intestinal tract from self-digestion and multiple microorganisms [12]. MUC5AC forms a protective gel on the surface of the gastric mucosa, and it increases in the intestinal tract during infection [13]. TFF3, a type of peptide, plays an important role in protecting and repairing the epithelium [14]. Compared with the control group, the LPS group showed a significant decrease in MUC2 in the ileum tissue (*p* < 0.05, Figure 4A), while MUC5AC significantly increased (*p* < 0.01, Figure 4B) and the content of sIgA decreased, indicating that LPS caused the dysfunction of the ileum mucus layer. After the oral administration of SGE, the secretion of MUC2 significantly increased (*p* < 0.05, Figure 4A). In contrast, the secretion of MUC5AC significantly decreased (*p* < 0.01, Figure 4B). The levels of trefoil factor TFF3 (*p* < 0.01, Figure 4C) and sIgA significantly increased (*p* < 0.05, Figure 4D), indicating that the function of the ileum mucus layer improved after SGE treatment.

### 2.5. Effect of SGE on the Ileitis Factors in Mice

**Figure 5:**
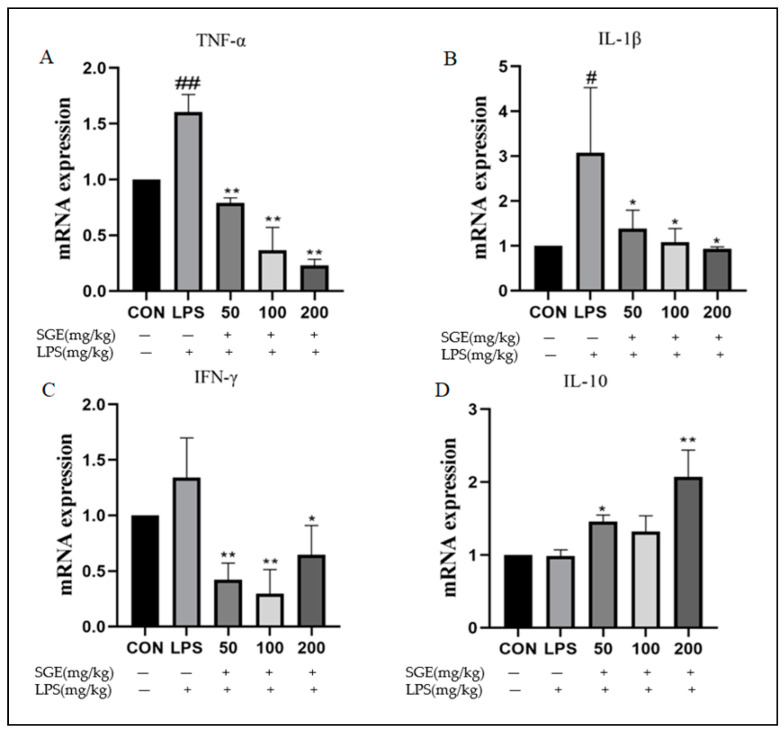
SGE improved LPS-induced intestinal inflammation in mice. Fluorescence quantitative real-time polymerase chain reaction was used to detect the expression of ileitis factors: TNF-α (**A**), IL-1β (**B**), IFN-γ (**C**), and IL-10 (**D**) mRNA. (*n* = 6). Compared with the CON group, # *p* < 0.05, ## *p* < 0.01. Compared with the LPS group, * *p* < 0.05, ** *p* < 0.01.

Compared with the control group, LPS significantly increased the expression of the inflammatory factors TNF-α (*p* < 0.01, Figure 5A), IL-1β (*p* < 0.05, Figure 5B), and IFN-γ (Figure 5C) mRNA in mouse ileum tissue. Compared with the LPS group, SGE significantly inhibited TNF-α (*p* < 0.01, Figure 5A), IL-1β (*p* < 0.05, Figure 5B), and IFN-γ (*p* < 0.01, Figure 5C) and increased the expression of the anti-inflammatory factor IL-10 (*p* < 0.05, Figure 5D). These results show that SGE restored intestinal function by regulating inflammatory factors.

### 2.6. Effects of SGE on INOS and CD206 Protein in Ileum

**Figure 6:**
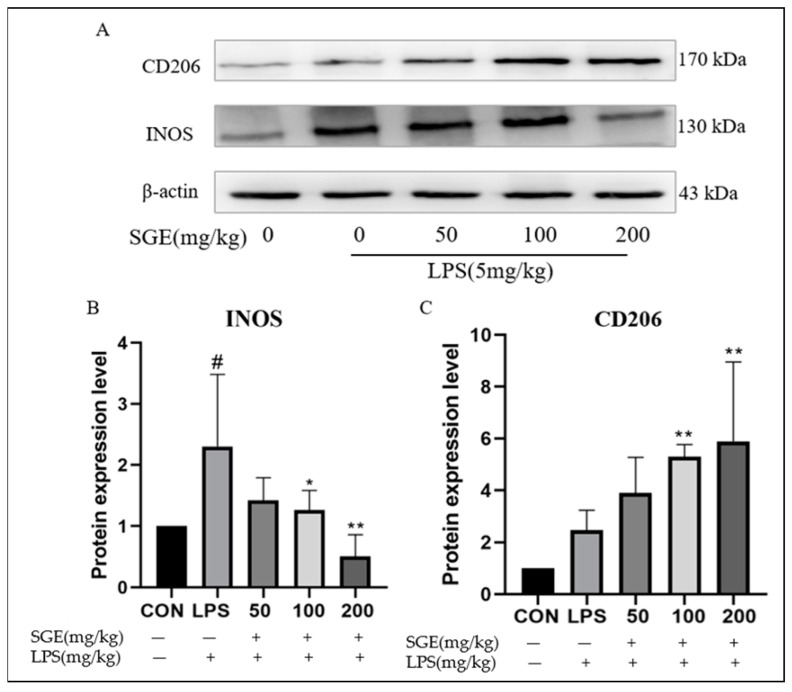
Effects of SGE on the protein expression of iNOS and CD206 in the ileum. Western blotting of INOS and CD206 proteins in mouse ileum (**A**). The effect of SGE on INOS (**B**) and CD206 (**C**) protein expression levels (*n* = 3). Compared with the control group, # *p* < 0.05. Compared with the LPS group, * *p* < 0.05, ** *p* < 0.01.

The protein blot results are shown in Figure 6A. Compared with the CON group, LPS significantly upregulated the expression of INOS in the ileum (*p* < 0.05, Figure 6B). Compared with the LPS group, SGE significantly decreased the expression of INOS (*p* < 0.05, Figure 6B) while significantly increasing the expression of CD206 (*p* < 0.01, Figure 6C). These results indicate that ileal inflammation was significant after LPS stimulation, while SGE treatment alleviated intestinal inflammation.

### 2.7. Cytotoxicity of SGE and Its Application in Inflammatory Model

**Figure 7:**
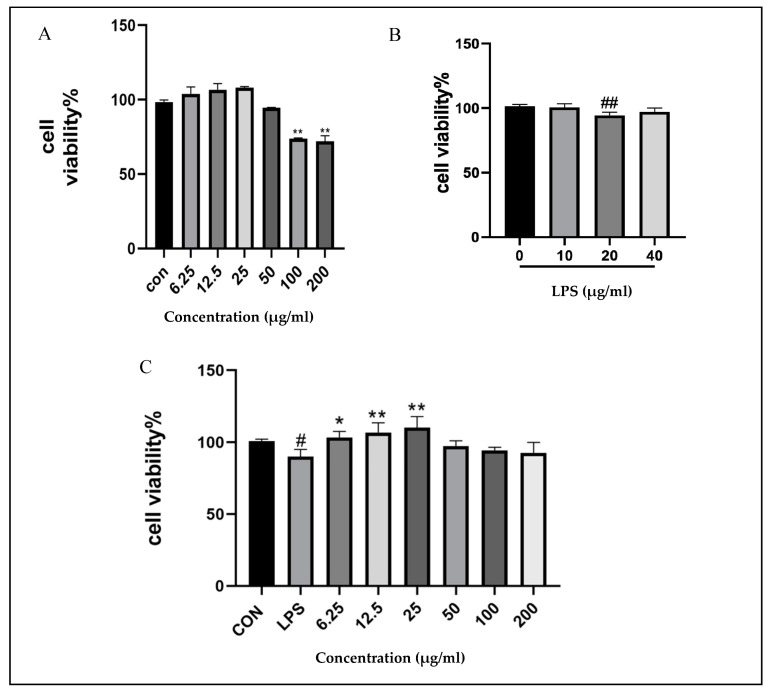
Cytotoxicity of SGE on LPS-stimulated RAW264.7 cells. The effect of SGE on the cytotoxicity of RAW264.7 cells (**A**). Screening of LPS model (**B**) viability of RAW264.7 cells stimulated by LPS at different concentrations of SGE (**C**) (*n* = 3). Compared with the control group, # *p* < 0.05, ## *p* < 0.01. Compared with the LPS group, * *p* < 0.05, ** *p* < 0.01.

The effect of SGE on the activity of RAW264.7 cells was detected. Figure 7 shows that when exposed to 100 μg/mL and 200 μg/mL SGE, cell viability significantly decreased (*p* < 0.01, Figure 7A) compared with the control group. Different concentrations of LPS were used to treat RAW264.7 cells. Compared with the control group, 20 μg/mL LPS treatment significantly reduced the activity of RAW264.7 cells (*p* < 0.01, Figure 7B). RAW264.7 cells were treated with different concentrations of SGE for 12 h, followed by LPS at a concentration of 20 μg/mL for 9 h, to establish a RAW264.7 cell inflammation model. Compared with the LPS group, SGE treatment at concentrations of 6.25 μg/mL (*p* < 0.05), 12.5 μg/mL (*p* < 0.01), and 25 μg/mL (*p* < 0.01) significantly increased cell viability (Figure 7C). Therefore, 6.25, 12.5, and 25 μg/mL were used as the standards for low, medium, and high concentrations of SGE.

### 2.8. Effects of SGE on the Inflammatory Factors in RAW264.7 Cells

**Figure 8:**
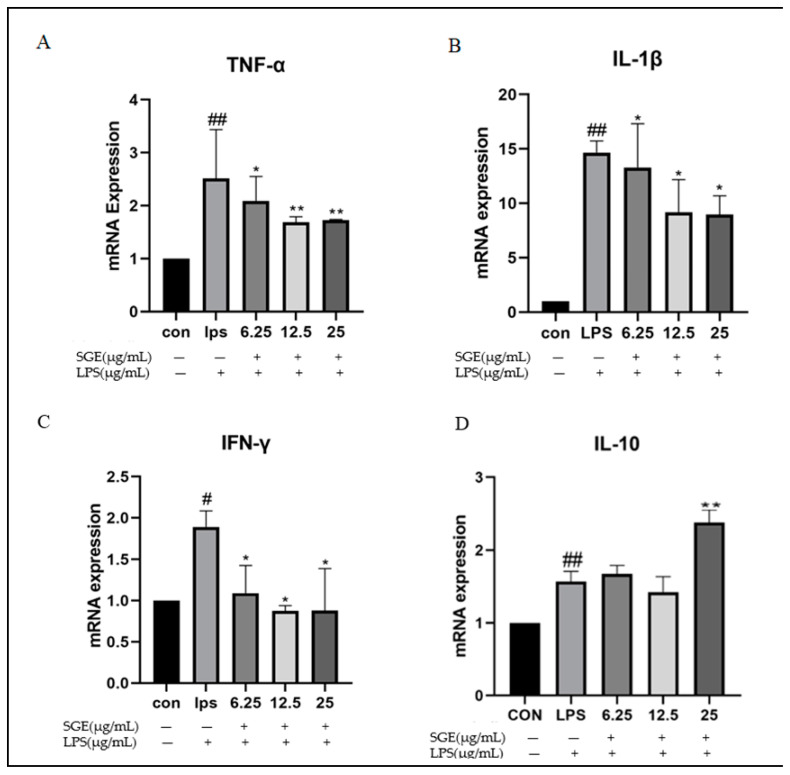
Effects of SGE on the regulation of inflammation in RAW264.7 cells. Detection of pro-inflammatory cytokines in RAW264.7 cells with RT-qPCR: TNF-α (**A**), IL-1β (**B**), IFN-γ (**C**), and the expression of the anti-inflammatory cytokine IL-10 (**D**) mRNA (*n* = 3). Compared with the control group, # *p* < 0.05, ## *p* < 0.01. Compared with the LPS group, * *p* < 0.05, ** *p* < 0.01.

The inflammatory factors in mouse RAW264.7 cells were detected using RT-qPCR, and the results are shown in Figure 8. Compared with the control group, LPS significantly increased the expression of inflammatory cytokines in RAW264.7 cells: TNF-α (*p* < 0.01, Figure 8A), IL-1β (*p* < 0.01, Figure 8B), and IFN-γ (*p* < 0.05, Figure 8C). Compared with the LPS group, SGE significantly inhibited the expression of TNF-α (*p* < 0.05, Figure 8A), IL-1β (*p* < 0.05, Figure 8B), and IFN-γ (*p* < 0.05, Figure 8C), while increasing the expression of the anti-inflammatory factor IL-10 (*p* < 0.01, Figure 8D).

### 2.9. Effects of SGE on RAW264.7 Macrophage Polarization

**Figure 9:**
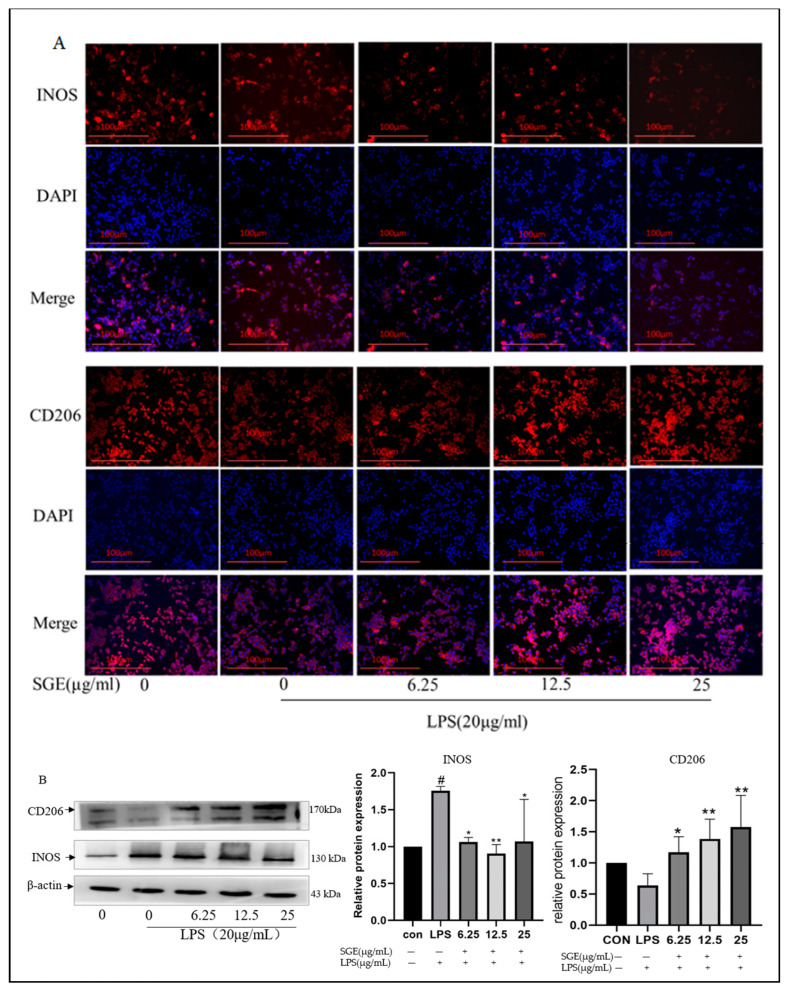
Effect of SGE on RAW264.7 cell polarization. The fluorescence intensity of INOS and CD206 proteins was detected with immunofluorescence(**A**), and the expression of INOS and CD206 proteins was detected with Western blotting (**B**) (*n* = 3). Compared with the control group, # *p* < 0.05. Compared with the LPS group, * *p* < 0.05, ** *p* < 0.01.Note: Inducible nitric oxide synthase (INOS) in the red fluorescent group represents M1 macrophages; CD206 represents M2 macrophages in the red fluorescent group; the nucleus was stained with DAPI (blue fluorescent clusters).

As shown in Figure 9, the fluorescence intensity of macrophage M1 and macrophage M2 markers in RAW264.7 cells was detected with immunofluorescence. The results showed that, compared with the control group, the fluorescence intensity of INOS increased after LPS treatment (Figure 9A), while the fluorescence intensity of CD206 decreased (Figure 9A). The immunoblot results showed a significant increase in INOS (*p* < 0.05, Figure 9B). Compared with the LPS group, the fluorescence intensity of INOS decreased, and the fluorescence intensity of CD206 increased after SGE treatment (Figure 9A). The immunoblotting results also showed a significant decrease in INOS protein expression (*p* < 0.05, Figure 9B) and a significant increase in CD206 protein expression (*p* < 0.05, Figure 9B). These results indicate that SGE regulates the polarization of macrophages to the M2 type and plays an anti-inflammatory role.

## 3. Discussion

The primary constituents of SGE were determined through LC-MS/MS analysis, which is commonly used for the qualitative and quantitative analysis of pharmaceuticals [15]. The main constituents of SGE include fatty acids, organic acids, sugars, and triterpenoid glycosides. Mogroside, a triterpenoid glycoside, is used as a quality standard for measuring *Siraitia grosvenorii* in the Pharmacopoeia of the People’s Republic of China. Mogroside V is known to have a significant chemopreventive effect on inflammatory lesions in LPS-stimulated RAW264.7 cells [16]. In this study, the relative content of mogroside in SGE was 1.27%, which meets the quality standard of *Siraitia grosvenorii* (relative content of mogroside ≥ 0.5%) in the Pharmacopoeia of the People’s Republic of China. Therefore, SGE could be used as a form of traditional Chinese medicine formulated as granules for clinical treatment. It is known that purified homogeneous polysaccharides isolated from the roots of *Siraitia grosvenorii* have immunomodulatory activity [17]. Manipulating the composition of dietary fatty acids, which play an important role in epithelial inflammatory reactions and mucosal immune responses, can influence the gut health of piglets [18]. Citric acid, a type of organic acid, can fortify the intestinal tight junction barrier and boost intestinal immune function in mice [19]. Thus, the main ingredients of SGE, including mogroside, sugars, fatty acids, and organic acids, all play critical roles in regulating inflammation.

The intestine maintains tissue homeostasis by resisting foreign invasion through the immune defense mechanism of the mucosal barrier [20]. The intestinal mucosal barrier includes secreted immunoglobulin (sIgA) and an intestinal mucus layer, which is primarily composed of mucin secreted by goblet cells and epithelial cells [21,22,23]. The dysfunction of the mucus layer is a critical initial event in the pathogenesis of intestinal diseases such as inflammatory bowel disease [24]. When the mucosal barrier is damaged, pathogenic microorganisms can enter the bloodstream, causing systemic immune suppression and weight loss. LPS challenges can damage intestinal epithelial integrity and disrupt the barrier functions of the gastrointestinal tract [25]. Previous research has shown that LPS induces an inflammatory response and damages the normal physiological function in the jejunal epithelial cells of piglets [26]. LPS is widely used to establish models of inflammation [27,28]. The ileum contains a specialized lymphoid structure called Peyer’s patch, which plays a central role in the intestinal mucosal immune response and immune induction [20,29]. In this study, LPS damaged intestinal morphology, significantly decreased the ratio of intestinal villus length to crypt depth, reduced the number of goblet cells, and decreased the mRNA expression of MUC2 in the ileum. These findings indicate that LPS damaged the intestinal structure and disrupted the normal function of the intestinal mucosa. Additionally, LPS treatment altered blood indices (EOS%, NEU%, and the number of white blood cells) and decreased the content of IgG in the serum, serving as characteristic indicators of systemic inflammatory infection [29,30,31]. This confirms that LPS induced systemic inflammation. The oral administration of SGE protected intestinal morphology, significantly increased the ratio of intestinal villus length to crypt depth, increased the number of goblet cells, increased the mRNA expression of MUC2 and TFF3 in the ileum, and elevated the content of sIgA in the ileum and IgM and IgG in the serum. These results indicate that SGE blocked systemic inflammation by maintaining the integrity and function of the intestinal mucus layer, which also explains why the body weights of the mice in the SGE groups were higher than those of the control group.

Pathogens escaping from the mucosal layer are detected and recognized by the innate immune system, with macrophages playing a crucial role in defense. Their phenotype and function often vary depending on the inflammatory state and resolution of inflammation [32]. When induced by bacterial LPS, M1 macrophages produce pro-inflammatory cytokines such as interleukin-6 (IL-6), interleukin-1β (IL-1β), and tumor necrosis factor α (TNF-α) [33]. In contrast, M2 macrophages, induced by interleukin-4 (IL-4), produce anti-inflammatory cytokines, including arginase-1 (Arg-1) and interleukin-10 (IL-10) [34]. The expression of inducible nitric oxide synthase (iNOS), an indicator of inflammation severity, increases when macrophages polarize to the M1 type, while M2 macrophages, marked by CD206, promote tissue repair [35,36,37]. In this study, LPS treatment significantly upregulated iNOS protein expression and pro-inflammatory factor (IL-1β, TNF-α) mRNA expressions in vivo and in vitro, indicating that LPS induced macrophage polarization to the M1 type. Additionally, the oral administration of SGE induced a shift from M1 to M2 macrophage polarization, which is believed to be the critical reason why SGE alleviated intestinal inflammation and maintained the integrity and function of the intestinal mucus layer.

It can be seen that when the mice were given SGE at a dose of 50 mg/kg, SGE protected the intestinal structure and reduced the expression of inflammatory factors in the ileum, but SGE did not significantly change subsequent indicators such as peripheral blood WBC and NEU counts, serum sIGA levels, ortheprotein expression of iNOS and CD206 in the ileum. When administered to the mice at doses of 100 or 200 mg/kg, SGE exerted a broad regulatory function. Therefore, the recommended concentration of SGE in mice was 100 mg/kg. SGE can protect against heat-stress-induced intestinal damage and alleviate lung tissue damage in a chronic obstructive pulmonary disease murine model [38,39]. However, the dose of SGE used for anti-inflammatory effects in heat-stress-induced intestinal damage was as high as 600 mg/kg [39], much higher than our recommended concentration in this study. The yield of dried SGE used to protect lung tissue in the concentration range of 50–200 mg/kg was about 5% [38]. In this experiment, the yield of SGE was about 24%, indicating that our extraction process was more efficient, with extraction times and alcohol purity being key factors.

## 4. Materials and Methods

### 4.1. Material

*Siraitia grosvenorii* was purchased from Hesheng Pharmacy Co., Ltd. (Zhanjiang, China). LPS was obtained from Sigma Aldrich (St. Louis, Missouri, USA). The fixation and staining reagents were sourced from a biological company. Fetal bovine serum was purchased from ZETA LIFE Company (Paris, France), while penicillin, streptomycin, and trypsin were obtained from GIBCO Company (Los Angeles, California, USA) in the United States. DMEM high-glucose medium, fixation, and staining reagents were purchased from biological companies. Antibodies against iNOS, CD206, and Alexa Fluor 647-conjugated mouse IgG were purchased from Abcam Company (Cambridge, UK). The ECL chemiluminescence assay kit and DAPI were obtained from YEASEN Company (Shanghai, China). Mouse IgG, IgM, and sIgA kits were provided by Jiangsu Enzyme Immunoassay Industry Co., Ltd. (Nanjing, China).

### 4.2. Preparation of SGE

Dried *S. grosvenorii* fruit was extracted with water. The residue was extracted with water two more times, and the extracts were combined. This mixture was then extracted with 80% ethanol (*v*/*v*). The extract was filtered, concentrated, freeze-dried, and stored at 4 °C. The extraction yield after drying was 23.8% (*w*/*w*).

### 4.3. Analysis of Chemical Constituents of SGE

Here, 1 g of freeze-dried SGE was added to 500 microliters of methanol, sonicated for 30 min at 4 °C, and centrifuged for 10 min at 13,000 rpm, and the supernatant was used for detection (LCMS). The LCMS conditions were as follows: a flow rate of 0.3 mL/min and an injection volume of 10 μL. The mobile phase consisted of 0.1% formic acid/acetonitrile (B) and 0.1% formic acid/water (A). The gradient elution program is shown in Table 2. The LCMS data were automatically checked against databases (including ChemSpider, CHEBI, CHEMBL, natural product database, flavonoid database, OTC traditional Chinese medicine database, and mzCloud) using Compound Discoverer software V 3.2 ( Thermo Fisher Scientific, Waltham, MA, USA).

### 4.4. Animal Experiment

Thirty male C57/BL6 mice, purchased from Guangzhou Furuoge Biotechnology Co., Ltd.(Guangzhou, China), were housed in an SPF-grade facility at 22 ± 3 °C with a 12/12 h light/dark cycle, with free access to food and water. After one week of adaptation, the experiment began. The mice were randomly divided into five groups (*n* = 6): the control group (given PBS by gavage), the LPS model group (given PBS by gavage and 5 mg/kg LPS intraperitoneally on the last day), the low-dose SGE group (50 mg/kg SGE by gavage), the medium-dose SGE group (100 mg/kg SGE by gavage), and the high-dose SGE group (200 mg/kg SGE by gavage) for 14 days. On the 15th day, the LPS model group and SGE groups received an intraperitoneal injection of 0.2 mL (5 mg/kg) LPS. The reagent dosages used were based on the literature [40]. Body weight and food intake were recorded throughout the experiment. Six hours after the LPS injection, the mice were euthanized, and blood and ileal tissue samples were collected.

### 4.5. Routine Analysis of Blood

After collecting whole blood from the mice, the number of white blood cells, the percentage of neutrophils, and the percentage of eosinophils were measured using a blood routine analyzer.

### 4.6. ELISA

According to the manufacturer’s instructions, kits from Jiangsu Enzyme-linked Immunoassay Industry Co., Ltd. (Nanjing, China) were used to measure the concentrations of serum IgG, IgM, and ileal sIgA.

### 4.7. HE and PAS Staining

Mouse ileal tissue was fixed in 4% paraformaldehyde, embedded in paraffin, sectioned, and stained with hematoxylin and eosin (HE) and periodic acid Schiff’s (PAS) stain, with 6 samples per group. Histopathological changes in the tissues were evaluated using an optical microscope (Olympus, Tokyo, Japan). The length of intestinal villi, depth of crypts, and relative number of goblet cells were measured with Image Pro Plus.

### 4.8. Cell Culture and Model Establishment

The mouse macrophage cell line RAW264.7, preserved in the laboratory, was cultured in DMEM medium containing 10% fetal bovine serum, 100 μg/mL streptomycin, and 100 U/mL penicillin in an incubator with 5% CO_2_ at 37 °C. The cells were treated with different concentrations of SGE (0, 6.25, 12.5, 25, 50, 100 μg/mL) for 12 h. Then, 10 μL of CCK-8 solution was added, and the cells were incubated for 1 h. Absorbance at 450 nm was measured with an enzyme-labeling instrument to assess cell viability. To detect cell viability with LPS, 100 μL of 0, 10, 20, and 40 μg/mL LPS solution was added to a 96-well culture plate and cultured for 9 h. Then, 10 μL of CCK-8 solution was added, incubated for 1 h, and absorbance at 450 nm was measured with an enzyme-labeling instrument.

### 4.9. RTq-PCR

According to the manufacturer’s instructions, total RNA was isolated from treated cells and quick-frozen ileum samples using an ultra-pure total RNA extraction kit (Invitrogen, Waltham, MA, USA). The concentration and purity of total RNA were measured at 260 and 280 nm with an OD1000 spectrophotometer. The reaction system and procedures were configured according to the instructions of the Universal Blue qPCR SYBR Master MIX kit. Data were analyzed using the 2^−ΔΔCt^ method and calculated with β-actin as a standardized control. The primers for RT-qPCR (Table 3) were synthesized by Shenggong Biological Co., Ltd. (Shanghai, China).

### 4.10. Western Blot

According to the manufacturer’s instructions, protein was extracted from RAW264.7 cells and ileum tissue using RIPA lysis buffer (P0013B, Beyotime, China). The protein was quantitatively analyzed with the BCA protein detection kit (KGP1100, KeyGEN). Equal amounts of protein were separated on a polyacrylamide gel, transferred to a polyvinylidene fluoride membrane, and then blocked for at least 30 min before being incubated with the primary antibody overnight at 4 °C. The following antibodies were used: INOS, CD206, and β-actin, all at a 1:1000 dilution. The secondary antibody included goat anti-rabbit antibody labeled with horseradish peroxidase (HRP) and a 1:1000 diluted anti-mouse IgG (Quan-type Jin, Beijing, China). The membrane was then incubated with the secondary antibody for 2 h. The protein bands were detected using chemiluminescence, and the protein blots were quantified using Image J (v1.8.0).

### 4.11. Immunofluorescence

RAW264.7 cells were seeded in 24-well plates, treated with SGE, washed three times with PBS, fixed with 4% paraformaldehyde for 30 min, permeabilized for 10 min, and washed three times with 1% BSA. The cells were then blocked with 5% BSA for 30 min, incubated with the primary antibody overnight at 4 °C, washed three times with PBS, and incubated with rabbit IgG antibody coupled with biotin for 2 h in the dark. After three more washes with PBS, an anti-fluorescence quencher containing DAPI was added. Fluorescence imaging was performed using a fluorescence microscope.

### 4.12. Statistic Analysis

All experiments were repeated at least three times, and statistical significance was determined using one-way ANOVA, t-test, and LSD multiple comparisons. A difference was considered statistically significant when *p* < 0.05. All statistical analyses were conducted using IBM SPSS 23.0 statistical software, and all bar and line charts were created with GraphPad Prism 8.0.

## 5. Conclusions

**Figure 10:**
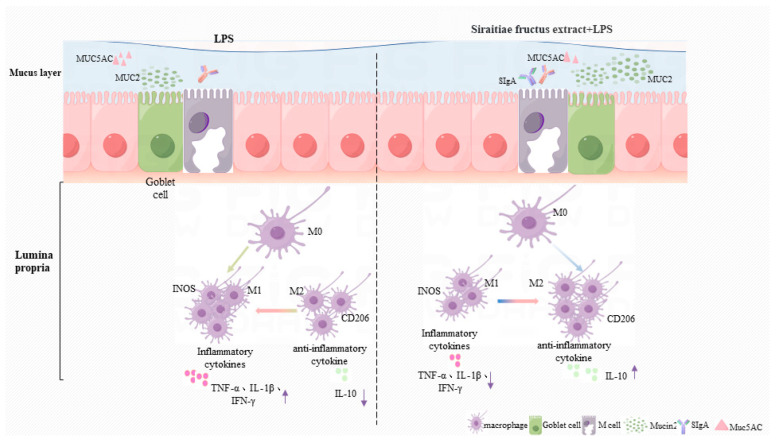
Protective mechanism of SGE on LPS-induced enteritis in mice.

In summary, SGE protected the mucus barrier, alleviated intestinal inflammation, and blocked the systemic immunosuppressive response by increasing M2 macrophages (Figure 10), providing a basis for further understanding the treatment of intestinal inflammation.

## Figures and Tables

**Table 1:** Chemical composition and relevant information of SGE.

Mode	Chemical Compound	Molecular Formula	Molecular Weight	Retention (min)	Peak Area	Peak Area Percentage %
P	Oleamide	C18H35NO	281.27138	16.168	4.90 × 10^10^	19.20
P	Hexadecanamide	C16H33NO	255.25575	16.002	2.11 × 10^10^	8.28
P	Stearamide	C18H37NO	283.28706	17.444	1.69 × 10^10^	6.61
P	N-Methylanthranilic acid	C8H9NO2	151.06336	5.082	1.03 × 10^10^	4.05
P	Erucamide	C22H43NO	337.33409	13.409	9.96 × 10^9^	3.90
P	Palmitic acid	C16H32O2	273.26619	10.283	6.70 × 10^9^	2.62
P	3-Aminophenol	C6H7NO	109.0528	1.149	4.36 × 10^9^	1.71
P	Choline	C5H13NO	103.09977	0.99	4.02 × 10^9^	1.57
P	Trigonelline HCl	C7H7NO2	137.04791	1.033	3.63 × 10^9^	1.42
P	3-Hydroxy-2-methylpyridine	C6H7NO	109.05282	4.957	2.97 × 10^9^	1.16
N	DL-Malic acid	C4H6O5	134.02132	1.074	4.33 × 10^10^	16.93
N	Citric acid	C6H8O7	192.02679	1.166	1.46 × 10^10^	5.72
N	D-(+)-Glucose	C6H12O6	226.06866	1.245	9.63 × 10^9^	3.77
N	L-Threonic acid	C4H8O5	136.03703	0.888	4.34 × 10^9^	1.70
N	Gluconic acid	C6H12O7	196.05797	1.012	2.92 × 10^9^	1.14
N	Mogroside	C60H102O29	1286.64704	6.4–8.2	4.66 × 10^8^	1.27

**Table 2:** Mobile-phase gradient elution procedure.

Time (min)	A (0.1% FA/Water)	B (0.1% FA/ACN)
0	90	10
10	0	100
15	0	100
17.1	90	10
20	90	10

**Table 3:** Primers used for real-time fluorescence quantitative PCR.

Gene	Primer Sequence (5′ to 3′)	Login Number	Prodsize
β-actin	F: ACTGCCGCATCCTCTTCCTC	NC_000071.7	80
	R: AACCGCTCGTTGCCAATAGTG		
TNF-α	F: CGCTCTTCTGTCTACTGAACTTCGG R: GTGGTTTGTGAGTGTGAGGGTCTG	NC_000083.7	113
IL-1β	F: TCGCAGCAGCACATCAACAAG R:TCCACGGGAAAGACACAGGTAG	NC_000068.8	94
IFN-γ	F: CTGGAGGAACTGGCAAAAGGATGG R: GACGCTTATGTTGTTGCTGATGGC	NC_000076.7	121
IL-10	F:GGACAACATACTGCTAACCGACTCC R: AGCCGCATCCTGAGGGTCTTC	NC_000067.7	186
MUC2	F:GCTGACGAGTGGTTGGTGAATG R:GATGAGGTGGCAGACAGGAGAC	NC_000073.7	135
MUC5AC	F:TCACTCTACCACTCCCTGCTTCTG R:CACCTGACAATCCTGGCTACACATC	NC_000073.7	127
TFF3	F:AATGCTGTTGGTGGTCCTGGTTG R:GGGCACATTTGGGATACTGGAGTC	NC_000083.7	181

## Data Availability

The original data of the study are included in the Supplementary Material. Enquiries can be directed to the corresponding author(s).

## Supplementary Materials

The following supporting information can be downloaded at: https://www.mdpi.com/article/10.3390/ph17081023/s1. Table S1. Chemical constituents of *Siraitia grosvenorii* extract. Table S2. The data about mice body weight in five groups.
