# Supplementary material for: Siraitia grosvenorii Extract Protects Lipopolysaccharide-Induced Intestinal Inflammation in Mice via Promoting M2 Macrophage Polarization
Source: Pharmaceuticals (Basel). 2024 Aug 4;17(8):1023. doi: 10.3390/ph17081023 (PMC11357656; doi:10.3390/ph17081023)
Supplement: Supplementary file 1 [file pharmaceuticals-17-01023-s001.zip › Table S1 Chemical constituents of Siraitia grosvenorii extract.pdf]

mode	chemical compound	Molecular formula	molecular weight	Retention（min)	peak area	peak area percentage %
P	Oleamide	C18H35NO	281.27138	16.168	4.90E+10	19.20
P	Hexadecanamide	C16H33NO	255.25575	16.002	2.11E+10	8.28
P	Stearamide	C18H37NO	283.28706	17.444	1.69E+10	6.61
P	N-Methylanthranilic Acid	C8H9NO2	151.06336	5.082	1.03E+10	4.05
P	Erucamide	C22H43NO	337.33409	13.409	9.96E+09	3.90
P	Palmitic Acid	C16H32O2	273.26619	10.283	6.70E+09	2.62
P	3-Aminophenol	C6H7NO	109.0528	1.149	4.36E+09	1.71
P	Choline	C5H13NO	103.09977	0.99	4.02E+09	1.57
P	Trigonelline HCl	C7H7NO2	137.04791	1.033	3.63E+09	1.42
P	3-Hydroxy-2-methylpyridine	C6H7NO	109.05282	4.957	2.97E+09	1.16
P	Pyridoxal	C8H9NO3	167.05829	1.169	2.23E+09	0.87
P	L-Pyroglutamic acid	C5H7NO3	129.04268	1.255	2.04E+09	0.80
P	2,3,4,9-Tetrahydro-1H-β-carboline-3-carboxylic acid	C12H12N2O2	216.08998	4.497	1.34E+09	0.52
P	6-Hydroxyindole	C8H7NO	133.05276	5.018	1.33E+09	0.52
P	Bis(4-ethylbenzylidene)sorbitol	C24H30O6	414.20359	11.258	1.30E+09	0.51
P	cis,cis-Muconic acid	C6H6O4	142.02677	2.374	1.13E+09	0.44
P	Methylindole-3-acetate	C11H11NO2	189.07892	5.367	9.75E+08	0.38
P	Pipecolic acid	C6H11NO2	112.05248	0.995	8.81E+08	0.34
P	Chaulmoogric Acid	C18H32O2	297.26645	13.79	8.14E+08	0.32
P	Stachydrine	C7H13NO2	143.09473	1.138	7.30E+08	0.29
P	Berberine	C20H17NO4	335.11531	7.505	6.78E+08	0.27
P	3-(2-Hydroxyethyl)indole	C10H11NO	161.08399	7.21	4.66E+08	0.18
P	4-Methylumbelliferone	C10H8O3	176.04738	7.707	3.11E+08	0.12
P	1-Linoleoylglycerol	C21H38O4	336.26583	15.562	1.48E+08	0.06
P	Oleanolic acid	C30H48O3	438.34932	15.425	7.71E+07	0.03
P	3,5-di-tert-Butyl-4-hydroxybenzaldehyde	C15H22O2	234.16182	12.947	5.00E+07	0.02
P	Laurolactam	C12H23NO	180.15124	16.816	3.79E+07	0.01

P	CitroflexA-4	C20H34O8	402.22496	14.714	3.44E+07	0.01
P	L-Valine	C5H11NO2	117.07877	0.973	2.93E+07	0.01
P	Palmitoylethanolamide	C18H37NO2	299.28191	15.165	1.77E+07	0.01
P	Panthenol	C9H19NO4	205.13118	18.324	1.73E+07	0.01
P	Tridemorph	C19H39NO	297.30275	16.978	1.72E+07	0.01
P	α-Linolenic acid	C18H30O2	278.22426	12.734	1.55E+07	0.01
P	Ursolic acid	C30H48O3	456.35964	15.461	1.46E+07	0.01
P	α-Boswellic acid	C30H48O3	456.35994	7.662	1.39E+07	0.01
N	DL-Malic acid	C4H6O5	134.02132	1.074	4.33E+10	16.93
N	Citric acid	C6H8O7	192.02679	1.166	1.46E+10	5.72
N	(2R)-2,3-Dihydroxypropanoic acid	C3H6O4	106.02638	1.007	1.05E+10	4.11
N	D-(+)-Glucose	C6H12O6	226.06866	1.245	9.63E+09	3.77
N	Salsolinol	C10H13NO2	179.09438	8.456	4.88E+09	1.91
N	3-Hydroxy-3-(methoxycarbonyl)pentanedioic acid	C7H10O7	206.04255	1.591	4.55E+09	1.78
N	L-Threonic acid	C4H8O5	136.03703	0.888	4.34E+09	1.70
N	Gluconic acid	C6H12O7	196.05797	1.012	2.92E+09	1.14
N	δ-Ribono-1,4-lactone	C5H8O5	148.03699	1.043	2.44E+09	0.96
N	Mogroside V	C60H102O29	1286.64704	6.424	1.99E+09	0.78
N	δ-Gluconicacid δ-lactone	C6H10O6	178.04753	1.114	1.97E+09	0.77
N	Pyrogallol	C6H6O3	126.03162	1.894	1.50E+09	0.59
N	Sucrose	C12H22O11	342.11597	0.861	1.38E+09	0.54
N	Salicylamide	C7H7NO2	137.04754	3.326	9.05E+08	0.35
N	Ferulic acid	C10H10O4	194.05777	5.92	7.21E+08	0.28
N	Lactose	C12H22O11	388.1215	1.622	6.61E+08	0.26
N	Terephthalic acid	C8H6O4	166.02653	3.15	4.96E+08	0.19
N	mogroside IV	C54H92O24	1124.59623	6.808	4.18E+08	0.16
N	Mogroside Ⅲe/ⅢA1/ III/ III A2 or isomers	C48H82O19	962.54341	7.617	4.06E+08	0.16
N	Kojic Acid	C6H6O4	142.02655	1.759	3.74E+08	0.15

N	6-Hydroxypicolinic acid	C6H5NO3	139.02662	1.236	3.59E+08	0.14
N	Aconitic Acid	C6H6O6	174.01617	1.274	3.58E+08	0.14
N	4-Pyridoxic acid	C8H9NO4	183.05321	2.085	3.43E+08	0.13
N	6-Gingerol	C17H26O4	294.18394	10.502	2.48E+08	0.10
N	Leucine	C6H13NO2	131.09453	4.233	2.19E+08	0.09
N	Salicylic acid	C7H6O3	138.03152	7.164	1.97E+08	0.08
N	11-Oxomogroside V	C60H100O29	1284.63165	6.266	1.83E+08	0.07
N	L-Glutamic acid	C5H9NO4	147.05307	1.042	1.57E+08	0.06
N	Azelaic acid	C9H16O4	188.10481	6.761	1.47E+08	0.06
N	D-(+)-Mannose	C6H12O6	180.0632	11.397	1.34E+08	0.05
N	N-Acetylvaline	C7H13NO3	159.08945	4.694	1.18E+08	0.05
N	16-Hydroxyhexadecanoic acid	C16H32O3	272.23512	15.436	1.08E+08	0.04
N	N-Isovalerylglycine	C7H13NO3	159.08945	5.067	1.02E+08	0.04
N	Mogroside IIIe/IIIA1/ III/ III A2 or isomers	C48H82O19	962.54341	7.038	9.59E+07	0.04
N	Shikimic acid	C7H10O5	174.05252	1.676	9.40E+07	0.04
N	Mogroside IIE/ II B/ IIA/ II A2 or isomers	C42H72O14	799.4871	8.166	8.42E+07	0.03
N	Mogroside IIE/ II B/ IIA/ II A2 or isomers	C42H72O14	799.4871	7.89	8.42E+07	0.03
N	Resorcinol	C6H6O2	110.0367	3.852	7.26E+07	0.03
N	Glucose1-phosphate	C6H13O9P	260.02986	0.832	6.68E+07	0.03
N	3-Aminosalicylic acid	C7H7NO3	153.04257	3.731	6.41E+07	0.03
N	Benzyl6-O-beta-D-glucopyranosyl-beta-D-glucopyranoside	C19H28O11	478.16838	3.213	5.16E+07	0.02
N	4-Hydroxybenzaldehyde	C7H6O2	122.0367	4.984	5.03E+07	0.02
N	mogroside VI	C66H112O34	1448.70051	6.295	3.95E+07	0.02
N	9-hydroxy-10,12-octadecadienoic acid	C18H32O3	296.23497	12.91	3.89E+07	0.02
N	PYROCATECHUIC ACID	C7H6O4	154.02659	4.819	2.89E+07	0.01
N	(3beta,9xi,12beta,17xi)-20-{[6-O-(beta-D-Glucopyranosyl)-beta-D-glucopyranosyl]oxy}-12-hydroxydammar-24-en-3-yl2-O-beta-D-glucopyranosyl-beta-D-	C54H92O23	1154.60628	7.439	2.34E+07	0.01

	glucopyranoside					
N	Suberic acid	C8H14O4	174.08897	5.746	2.23E+07	0.01
N	Glycyrrhizic Acid,AmmoniumSalt	C42H62O16	822.40278	9.029	1.59E+07	0.01
N	4-Hydroxybenzoic acid	C7H6O3	138.03152	11.404	1.12E+07	0.00
N	Hosenkoside A	C48H82O20	1024.54414	8.056	1.07E+07	0.00
N	4-hydroxyphenylacetic acid	C8H8O3	152.04721	8.458	7.92E+06	0.00
N	Genistein	C15H10O5	270.05266	11.633	7.22E+06	0.00
