# Supplementary material for: Siraitia grosvenorii Extract Protects Lipopolysaccharide-Induced Intestinal Inflammation in Mice via Promoting M2 Macrophage Polarization
Source: Pharmaceuticals (Basel). 2024 Aug 4;17(8):1023. doi: 10.3390/ph17081023 (PMC11357656; doi:10.3390/ph17081023)
Supplement: Supplementary file 1 [file pharmaceuticals-17-01023-s001.zip › Table S2 The data about mice body weight in five groups.pdf]

		1	2	3	4	5	6
**Control group**	Day 1	22.24	20.65	21.16	20.92	20.38	20.91
	Day 2	22.14	20.10	20.48	20.70	21.67	21.37
	Day 3	22.11	20.19	21.76	20.60	21.06	21.41
	Day 4	22.54	20.54	21.58	21.06	22.26	21.95
	Day 5	22.24	20.82	21.17	21.76	22.59	22.33
	Day 6	22.20	21	21.33	21.49	22.23	21.85
	Day 7	22.20	20.88	21.14	21.93	22.49	22.45
	Day 8	22.06	20.89	22.05	21.67	22.64	22.1
	Day 9	22.22	20.88	22.06	21.72	22.93	22.21
	Day 10	22.14	20.73	21.63	22.02	23	22.08
	Day 11	21.3	20.69	21.75	21.53	22.84	22.15
	Day 12	22.14	21.1	22.14	22.09	22.49	22.62
	Day 13	22.35	21.15	22.21	22.3	22.94	22.69
	Day 14	22.85	21.19	22.3	22.25	23.22	23.11
	Day 15	22.68	21.5	22.6	22.21	23.52	23.2
**LPS group**	Day 1	20.83	20.47	20.36	20.58	22.8	21.34
	Day 2	21.09	21.22	20.39	22.63	20.52	21.27
	Day 3	21.34	20.62	21.05	22.63	21.07	21.02
	Day 4	21.62	21.03	21.34	20.84	22.52	21.30
	Day 5	21.44	20.97	21.35	22.83	21.60	21.66
	Day 6	21.43	21.40	21.26	23.08	21.87	22.16
	Day 7	21.66	21.18	21.17	22.74	21.42	21.97
	Day 8	21.85	21.04	20.99	23.01	21.42	22.8
	Day 9	21.93	21.34	21.3	23.4	21.42	22.88
	Day 10	21.92	21.28	21.51	22.78	21.36	22.16
	Day 11	22.1	21.48	21.86	23.85	21.7	22.71
	Day 12	22.21	21.51	21.27	23.31	22.12	22.81
	Day 13	22.48	21.77	21.27	23.6	22.24	23
	Day 14	22.68	21.79	21.61	23.38	22.17	23.07
	Day 15	22.98	21.44	20.68	21.73	21.25	20.82
**SGE 50mg/Kg**	Day 1	19.60	22.4	21.71	23.49	21.22	20.62
	Day 2	19.67	22.20	21.51	21.97	23.68	20.88
	Day 3	20.70	22.40	22.17	22.32	24.20	20.95
	Day 4	21.09	22.22	24.24	23.11	22.14	21.35
	Day 5	21.10	22.24	23.25	23.99	21.85	21.61
	Day 6	20.89	22.23	23.29	23.45	22.02	21.56
	Day 7	21.41	22.55	23.41	24.20	21.84	21.83
	Day 8	20.65	22.74	23.72	23.92	22.04	22.16
	Day 9	21.22	21.93	24.24	23.16	22.13	21.87
	Day 10	21.1	22.19	23.61	24.63	22.45	22.15
	Day 11	21.32	22.46	23.55	24.64	22.25	22.59
	Day 12	21.35	22.54	23.83	25	22.48	22.46

	Day 13	21.48	23	23.97	25.08	22.4	22.66
	Day 14	22.2	22.73	24.04	24.87	22.42	22.7
	Day 15	22.07	23.78	25.17	22.87	23.28	23.1
**SGE 100mg/Kg**	**Day 1**	20.61	21.99	21.07	21.09	21.40	21.28
	Day 2	21.18	22.06	21.70	21.31	21.54	21.80
	Day 3	21.55	22.30	21.90	21.70	21.60	21.50
	Day 4	21.04	22.09	21.25	21.23	21.62	21.45
	Day 5	21.36	22.34	21.25	21.72	21.65	21.91
	Day 6	21.11	22.66	21.74	21.85	21.74	22
	Day 7	21.17	22.93	22.22	21.86	22.85	22.08
	Day 8	21.36	23.1	21.74	22.48	22.49	22.76
	Day 9	21.71	22.89	22.07	22.82	22.45	22.62
	Day 10	21.18	23.33	22.3	22.92	23.36	22.92
	Day 11	21.47	23.07	22.37	23.42	22.62	22.93
	Day 12	21.45	22.98	22.33	22.9	22.37	22.69
	Day 13	21.55	23.17	22.64	23.51	23.13	22.87
	Day 14	21.52	23.08	22.29	23.53	23.03	22.78
	Day 15	21.85	23.5	23.8	22.48	23.09	23.37
**SGE 200 mg/Kg**	Day 1	20.67	22.09	20.21	22.8	20.71	21.12
	Day 2	20.59	22.71	23.13	21.31	20.82	21.95
	Day 3	20.53	23.07	22.98	21.04	20.49	21.59
	Day 4	20.30	22.95	20.77	22.68	20.57	21.82
	Day 5	20.58	22.80	22.90	21.35	20.35	21.28
	Day 6	20.35	22.69	22.99	21.68	20.51	21.63
	Day 7	20.60	22.84	22.73	21.35	20.12	21.29
	Day 8	20.85	23.81	23.31	21.93	20.99	21.56
	Day 9	20.59	23.69	23.48	21.3	21.01	21.6
	Day 10	20.4	23.59	23.2	21.6	20.74	21.55
	Day 11	20.61	24.33	23.38	21.96	21.05	21.55
	Day 12	20.77	24.24	24.23	22.42	21.19	21.76
	Day 13	20.86	24.21	23.75	22.44	21.78	21.93
	Day 14	20.98	24.6	24.72	22.33	21.56	22.07
	Day 15	20.98	24.7	24.08	22.65	21.88	21.81
